# Deferasirox Causes Leukaemia Cell Death through Nrf2-Induced Ferroptosis

**DOI:** 10.3390/antiox13040424

**Published:** 2024-03-29

**Authors:** Wan-Yi Hsu, Li-Ting Wang, Pei-Chin Lin, Yu-Mei Liao, Shih-Hsien Hsu, Shyh-Shin Chiou

**Affiliations:** 1 Division of Pediatric Hematology and Oncology, Department of Pediatrics, Kaohsiung Medical University Hospital, Kaohsiung Medical University, Kaohsiung 807, Taiwan; silverglassfish@gmail.com (W.-Y.H.); cooleylin@gmail.com (P.-C.L.); p920271a@gmail.com (Y.-M.L.); 2 Graduate Institute of Medicine, College of Medicine, Kaohsiung Medical University, Kaohsiung 807, Taiwan; 3 Department of Life Science, National Taiwan Normal University, Taipei 116, Taiwan; lisawang@ntnu.edu.tw; 4 Department of Pediatrics, School of Post-Baccalaureate Medicine, College of Medicine, Kaohsiung Medical University, Kaohsiung 807, Taiwan; 5 Center of Applied Genomics, Kaohsiung Medical University, Kaohsiung 807, Taiwan; 6 Department of Medical Research, Kaohsiung Medical University Hospital, Kaohsiung Medical University, Kaohsiung 807, Taiwan; 7 Research Center for Precision Environmental Medicine, Kaohsiung Medical University, Kaohsiung 807, Taiwan; 8 Graduate Institute of Clinical Medicine, College of Medicine, Kaohsiung Medical University, Kaohsiung 807, Taiwan

**Keywords:** deferasirox, BCP-ALL, Nrf2

## Abstract

Acute lymphoblastic leukaemia (ALL) is the most prevalent cancer in children, and excessive iron buildup resulting from blood transfusions and chemotherapy potentially has a negative impact on treatment outcomes and prognosis in patients with ALL. Therefore, initiating early iron chelation therapy during ALL treatment is a logical approach. Ideally, the selected iron chelator should also possess anti-leukaemia properties. The aim of the present study was to explore the potential impact and underlying mechanism of deferasirox (DFX) in ALL therapy. This study proved that DFX, an iron chelator, is capable of inducing leukaemia cell death through ferroptosis, which is achievable by increasing the expression of acetylated nuclear factor erythroid 2-related factor 2 (NRF2). More specifically, NRF2 acetylation on Lys599 was facilitated by acetyltransferase-p300/CBP. These findings indicate that DFX could serve as a potent adjunctive medication for patients with ALL. Moreover, DFX may offer dual benefits in ALL treatment, functioning as both an iron chelator and NRF2-modulating agent. Further research and clinical trials are necessary to fully elucidate the therapeutic potential of DFX in patients with ALL and incorporate it into treatment protocols.

## 1. Introduction

Acute lymphoblastic leukaemia (ALL) is the most common cancer in children. ALL is characterised by the uncontrolled proliferation of immature lymphoid cells [1]. Most patients with ALL respond well to chemotherapy. However, approximately 20–30% of patients still experience disease relapse [2,3]. A high bone-marrow iron content has been found to be associated with poor treatment response and identified as a risk factor of disease relapse [4,5]. Therefore, the identification of a novel adjuvant treatment is warranted.

Iron overload has long been recognised as a challenge in patients with leukaemia. Inevitable blood transfusions owing to chemotherapy-related cytopenia and the disease itself cause an excessive influx of iron into the body [6]. Furthermore, chemotherapy also induces an increase in non-transferrin-bound iron in the bloodstream [7,8]. Approximately 25% of paediatric leukaemia cases reportedly experience iron overload, which consequently has a negative impact on treatment response [9,10]. Excess iron participates in the Fenton reaction and causes ferroptosis, generating harmful reactive oxygen species (ROS) and impairing the normal haematopoietic process [11,12,13].

Ferroptosis is a unique and crucial type of programmed cell death that relies on iron and ROS [14]. Earlier studies inferred that ferroptosis participates in the biological functions of tumour suppression and immune surveillance [15]. Nonetheless, recent studies have increasingly revealed that ferroptosis also plays a role in pathological processes, such as cancer development, ischaemic heart disease, kidney failure, stroke, and liver damage [16,17].

Deferasirox (DFX), an iron chelator, is used clinically to alleviate the body’s iron burden, particularly in patients requiring chronic blood transfusions [18]. As an oral iron-chelating agent, DFX binds free iron in the body and is subsequently excreted mainly through bile or, to a lesser extent, via urine. It forms a complex with iron molecules, allowing the body to efficiently excrete the iron through urine and faeces [19,20]. DFX mitigates excess-iron-induced organ damage [19]. However, the specific effects of DFX on human ALL cells remain unreported.

Nuclear factor erythroid 2-like 2 (NFE2L2, abbreviated as NRF2) is a master transcription factor that promotes cellular antioxidant activity [21,22]. Normally, NRF2 is bound by two Kelch-like ECH-associated protein 1 (KEAP1) proteins in the cytoplasm. KEAP1 ubiquitinates NRF2, thereby marking it for proteasomal degradation. During oxidative stress, such as ROS, heavy metal, or nitric oxide abundance, KEAP1 is inactivated [22]. NRF2 is thus freed and enters the nucleus where it binds to antioxidant responsive elements (AREs) to express cytoprotection-related genes [23]. Phosphorylation and acetylation of NRF2 can also affect its binding ability with AREs [24]. In cancers, studies show that high NRF2 protects cells against high endogenous ROS, increases cellular resistance to chemotherapy, and reprograms cell metabolism toward proliferation [25]. In a study of patients with low-risk MDS [26], NRF2 expression was downregulated in peripheral blood cells after DFX treatment. There have been no reports of its activity in ALL. The aim of the present study was to investigate the potential impact and underlying mechanism of DFX in ALL therapy. Thus, we designed experiments to study whether DFX has a cytotoxic effect on leukaemia cells. Our results showed that DFX treatment leads to increased ROS production in cells from both patients with ALL and the leukaemia cell line, promoting cell death by enhancing NRF2 activity in ALL cells. Our findings suggest that DFX is a potent adjuvant therapy for patients with ALL and should be used earlier in the leukaemia treatment course rather than added after iron overload has developed.

## 2. Materials and Methods

### 2.1. Cells from Patients, Cell Lines, and Culture

Sup-B15 (human B-ALL with BCR-ABL translocation [t(9;22)(q34;q11)]) and Molt-4 (human T-ALL) cell lines were purchased from American Type Culture Collection (Manassas, VA, USA). The cells were cultured in a 5% CO_2_, 37 °C humidified incubator. Molt-4 cells were cultured in RPMI-1640 (Gibco, Thermo Fisher Scientific, Waltham, MA, USA) with 10% foetal bovine serum. Sup-B15 cells were cultured in a modified eagle medium (Gibco, Thermo Fisher Scientific, Waltham, MA, USA). Leukaemia cells from 22 patients were obtained at their initial diagnosis day, mostly from bone marrow. These patients, 4 T-ALL and 18 B-ALL cases, were from our hospital. Informed consents were obtained.

### 2.2. Reagents and Antibodies

DFX was purchased from MedChemExpress (Monmouth Junction, NJ, USA). Ammonium ferric citrate (AFC) and N-acetyl-cysteine (NAC) were obtained from Merck Millipore (Burlington, MA, USA). NRF2 and heme oxygenase-1 (HO-1) antibodies were purchased from Genetex (GTX103322, GTX101147; Irvine, CA, USA). KEAP-1 and Ser344 phosphorylation on NRF2 (abbreviated as Phos-Ser344) antibodies were obtained from Thermofisher (PA5-99434, PA5-67520; Waltham, MA, USA). Lys599 acetylation on NRF2 (abbreviated as Ace-Lys599) antibody was purchased from Abbkine (ABP50139; Atlanta, GA, USA). B-Actin (A2066), erastin (571203-78-6), and ferrostatin-1 (347174-05-4) antibodies were purchased from Sigma–Aldrich. HRP Goat antimouse and antirabbit IgG antibodies (Allbio, Taichung, Taiwan) were used as secondary antibodies for western blot analysis. BIO (GSK-3 Inhibitor), EX-527 (SIRT1 inhibitor), and C646 (p300/CBP inhibitor) were purchased from Selleckchem (S7915, S1541, S7152; Houston, TX, USA).

### 2.3. Viability Assay

Cell viability was measured using an XTT assay kit and Cell counting kit 8 (WST-8/CCK8). Both were purchased from Abcam (Cambridge, UK). In the XTT assay, cells were seeded into 96-well plates. The vehicles and reagents for the experiments were added and incubated for various times. Then, the XTT reagent, prepared according to the manufacturer’s protocol, was added to each well. After incubating for 2 h, formazan production was measured using the absorbance at 450 nm via the Versamax microplate reader (Molecular Devices, Sunnyvale, CA, USA) using the Softmax Pro 6.5.1 software. In CCK8 assay, cells were seeded into 96-well plates. The vehicles and reagents for the experiments were added and incubated for various times. Then, WST-8 solution was added and incubated for 3 h at 37 °C. Then, formazan production was measured using the absorbance at 420 nm via the Versamax microplate reader (Molecular Devices, Sunnyvale, CA, USA) using the Softmax Pro 6.5.1 software. All experiments were repeated at least three times.

### 2.4. Apoptosis Assay

The Annexin V Apoptosis Detection kit, purchased from Bioscience (Franklin Lakes, NJ, USA), was used to determine the extent of cell apoptosis. Cells were seeded into six-well plates, and DFX or vehicle was added. After incubation for 0, 16, and 24 h, the cells were extracted and collected into microcentrifuge tubes. Annexin V binding buffer and FITC Annexin V were added according to the manufacturer’s instructions. After incubation for 15 min in the dark, flow cytometry (LSR II, Bioscience, Franklin Lakes, NJ, USA) was performed to analyse the degree of apoptosis.

### 2.5. Autophagy Assay

An autophagy detection kit was purchased from Enzo life sciences (Lausen, Switzerland). Cells were cultured with vehicles or specified reagents. A positive control and negative control were added as per product protocol. Cells then were washed with Dulbecco’s phosphate-buffered saline, centrifuged, and resuspended. Cyto-ID Green Detection Reagent was added. Flow cytometric analysis was done as per product protocol.

### 2.6. Intracellular ROS and Superoxide Measurement

A Cellular ROS/Superoxide detection assay kit was purchased from Abcam (Cambridge, UK). Cells suspended (Molt4: 2 × 10^6^ cells/2 mL, Sup-B15: 1 × 10^6^ cells/2 mL) after DFX treatment for various times were seeded into a V-bottom 96-well plate. The assay was performed according to the manufacturer’s protocol, and wells were designated as positive (Pyocyanin) and negative (NAC) controls. The plate was centrifuged at 300× *g* to pellet the cells. Without disrupting the cell pellet, the culture media was removed and washed with 200 μL of cell-based assay buffer. The plate was centrifuged at 300× *g* to pellet the cells. The assay buffer was removed, leaving behind a small amount, and 130 μL of ROS staining buffer was added to each well, in addition to 10 μL of NAC reagent to designated negative control wells. The plate was incubated for 30 min at 37 °C. Then, 10 μL of the Pyocyanin working reagent was added to designated positive control wells and incubated for an additional hour at 37 °C. The plate was centrifuged, ROS staining buffer was removed, and 100 μL of cell-based assay buffer was added. The plate was read using a VersaMax™ ELISA Microplate Reader (Molecular Devices, Sunnyvale, CA, USA). All experiments were repeated at least three times.

### 2.7. Western Blot Analysis

Cells were collected from the plate, washed with phosphate-buffered saline, and the cell pellets were collected. Protein extracts were prepared using a radioimmunoprecipitation assay cell lysis buffer (VWR Life Science AMRESCO, Radnor, PA, USA). Proteins were loaded onto sodium dodecyl sulfate polyacrylamide gels for electrophoresis and then transferred to polyvinylidene difluoride membranes. The membranes were blocked with 5% nonfat milk at room temperature for 30 min and incubated with primary antibodies [1:500 dilution, KEAP-1, NRF2, HO-1, Ace-Lys599 (acetylation of Lys599), Phos-Ser344 (phosphorylation of Ser344); 1:5000 dilution, actin] overnight at 4 °C on a shaker. After washing, secondary antibodies (1:500 dilution) were added and washed, and the membranes were developed via chemiluminescence. All experiments were repeated at least three times.

### 2.8. NRF2 shRNAi Transfection

pLKO-NRF2i (10 nM) in OptiMEM (200 μL) was mixed with Lipo2000 (2.5 μL) in OptiMEM (200 μL) for 10 min and then added to a 1.5 mL Sup-B15 cell suspension. Cells were seeded into six-well plates (approximately 2 × 10^5^ cells/well) and analysed at the indicated times. NRF2 shRNA (sequence: GAGGATGTACTGAGTAAAGAT) was constructed using the pLKO vector by the National RNAi core facility at Academia Sinica (Taiwan). OptiMEM, Lipofectamine RNAiMAX, and the Lipofectamine 2000 manual were obtained from Thermo Fisher Scientific (Waltham, MA, USA).

### 2.9. Statistical Analysis

The results were expressed as the mean or as the mean ± standard error of the mean. Differences between experimental groups were evaluated using an unpaired t-test. We used prism 7.0 software (GraphPad Software, San Diego, CA, USA) to perform the analysis. *p* < 0.05 was considered statistically significant.

## 3. Results

### 3.1. DFX-Induced Programmed Cell Death in ALL Cells

**Figure 1:**
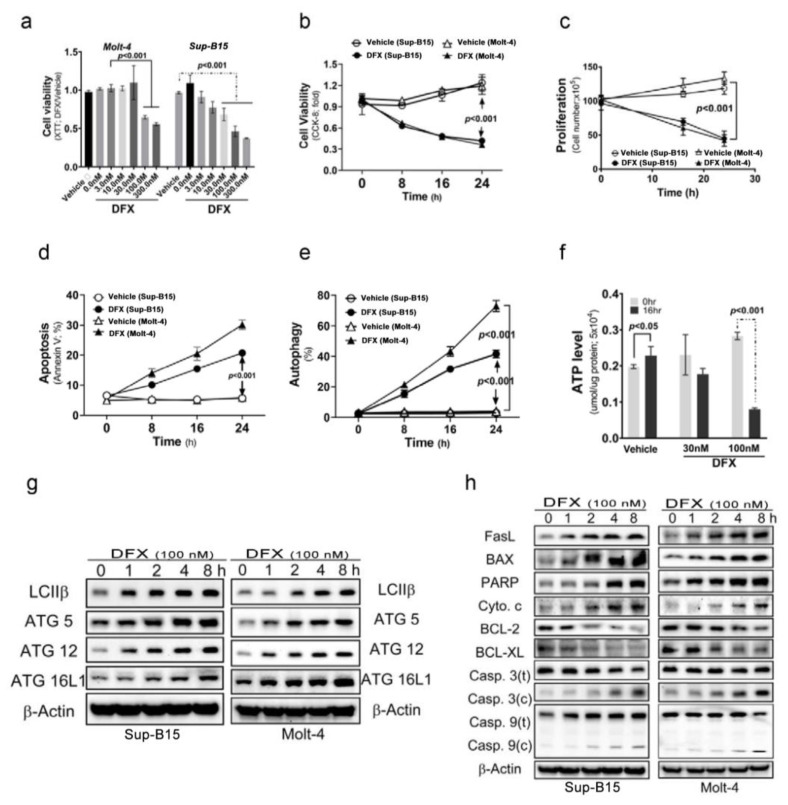
Deferasirox (DFX) causes acute lymphoblastic leukaemia (ALL) cell death. (**a**) Various concentrations of DFX were added to the culture medium, and cell viability was measured using an XTT assay after 24 h. The optimal concentration for our experiments was determined to be 100 nM. (**b**) Cells were treated with 100 nM DFX, and cell viability was assessed using Cell Counting Kit-8 (CCK-8) at 0, 8, 16, and 24 h. (**c**) Cells were treated with 100 nM DFX, and cell counts were calculated at 16 and 24 h. DFX significantly suppressed cell growth at 16 h. (**d**) The percentage of apoptotic Sup-B15 and Molt-4 cells was determined after DFX treatment. (**e**) The percentage of autophagic Sup-B15 and Molt-4 cells was determined after DFX treatment. (**f**) Adenosine triphosphate levels were measured using an enzyme-linked immunosorbent assay in Sup-B15 and Molt-4 cells after DFX treatment. (**g**) Western blotting was conducted to assess the levels of autophagic markers (LC3B, ATG5, ATG12, and ATG16L1) in lysates of Sup-B15 and Molt-4 cells after DFX treatment. (**h**) Western blotting was performed to analyse the levels of apoptotic markers in lysates of Sup-B15 and Molt-4 cells after DFX treatment.

To investigate the cytotoxic effects of DFX on ALL cells, Sup-B15 and Molt-4 cells were treated with varying concentrations of DFX. The results, shown in Figure 1a, reveal a significant decrease in cell viability detected using Cell Counting Kit-8 (CCK-8) when DFX was administered at concentrations of 100 and 300 nM. Thereafter, a fixed concentration of 100 nM DFX was administered and the incubation time varied. The findings, displayed in Figure 1b–e, demonstrate that DFX not only reduced cell viability and proliferation but also stimulated two kinds of programmed cell death (apoptosis and autophagy) in leukaemia cells. The cytotoxic effects of DFX became more pronounced in a time-dependent manner. Notably, a higher percentage of DFX-induced autophagic cells was observed than that of apoptotic cells in both cell types. DFX-induced apoptotic and autophagic activities were further monitored using the levels of several programmed cell death markers. DFX administration significantly increased apoptotic and autophagic marker expression in both ALL cell types (Figure 1g,h and Figure S1a,b). Further, DFX-treated Sup-B15 cells displayed a significantly decreased adenosine triphosphate level than vehicle cells (Figure 1f).

### 3.2. DFX Treatment Increased ROS Production by Regulating NRF2 Activity in ALL Cells

**Figure 2:**
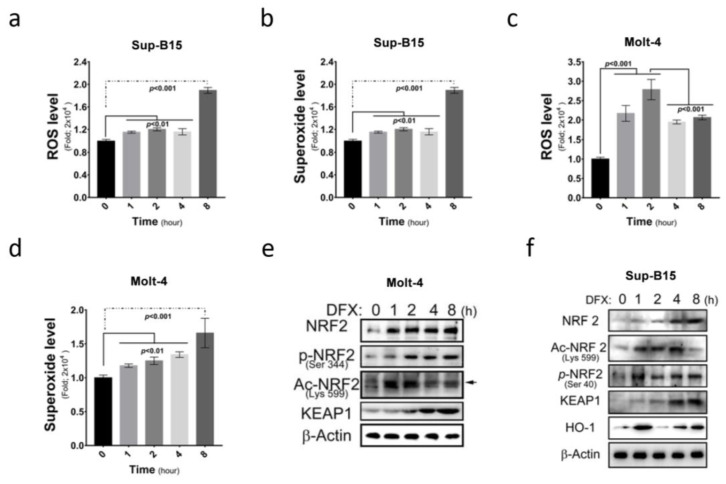
DFX increases intracellular reactive oxygen species (ROS) production and nuclear factor erythroid 2-related factor 2 (NRF2) expression in ALL cells. (**a**) Intracellular ROS levels were measured as fold increases in fluorescence relative to the negative control after 0, 1, 2, 4, and 8 h exposures of Sup-B15 cells to DFX treatment. (**b**) The fold change of superoxide levels was assessed after 0, 1, 2, 4, and 8 h exposures of Sup-B15 cells to DFX treatment. (**c**) Intracellular ROS levels were measured as fold increases in fluorescence relative to the negative control after 0, 1, 2, 4, and 8 h exposures of Molt-4 cells to DFX treatment. (**d**) The fold change of superoxide levels was assessed after 0, 1, 2, 4, and 8 h exposures of Molt-4 cells to DFX treatment. (**e**) Western blotting was conducted to assess the levels of NRF2, p-NRF2, Ac-NRF2, KEAP1, and HO-1 in lysates of Molt-4 cells after DFX treatment. (**f**) Western blotting was conducted to assess the levels of NRF2, p-NRF2, Ac-NRF2, KEAP1, and HO-1 in lysates of Sup-B15 cells after DFX treatment.

More and more medications are found to be capable of inducing ROS accumulation in cells. This study investigated ROS and superoxide levels in Sup-B15 and Molt-4 cells before and after treatment with DFX (deferasirox) and demonstrated a significant increase in total ROS and superoxide levels following DFX treatment in ALL cells (Figure 2a–d). Furthermore, this study examined the expression of NRF2, an ROS sensor protein, and its different active forms after DFX treatment. Elevated total NRF2 levels and different active NRF2 forms (phosphorylated in 344 a.a. and acetylated in 599 a.a.) were observed (Figure 2e,f and Figure S1c). These results demonstrate that DFX treatment significantly increased cellular ROS level and NRF2 expression. These results also correlated with the degree of cell death shown in Figure 1g,h.

### 3.3. DFX Induces Ferroptosis via the NRF2 Signalling Pathway in Leukaemia Cell Lines

**Figure 3:**
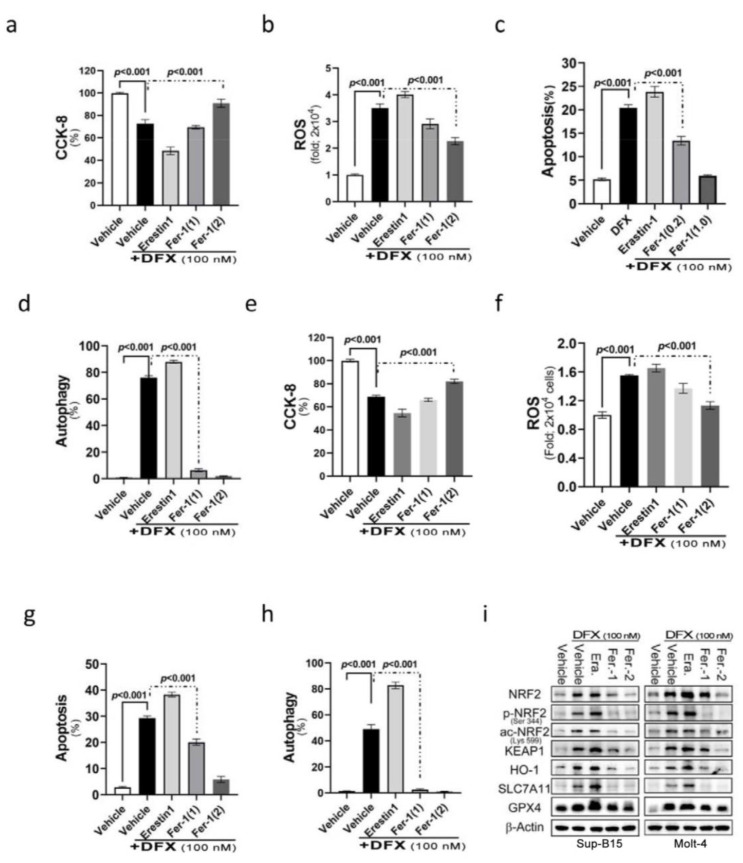
The NRF2 signalling pathway mediates DFX-induced ferroptosis in leukaemia cell lines. (**a**,**b**) Cell viability was evaluated using CCK-8 after treating Sup-B15 and Molt-4 cells with a combination of 100 nM DFX and either erastin or ferrostatin-1 for 24 h. (**c**,**d**) Following 24 h exposure of Sup-B15 and Molt-4 cells to a combination of 100 nM DFX and either erastin or ferrostatin-1, intracellular ROS levels were measured as fold increases in fluorescence relative to the negative control. (**e**,**f**) The percentage of apoptotic Sup-B15 and Molt-4 cells was determined after treatment with DFX and either erastin or ferrostatin-1. (**g**,**h**) The percentage of autophagic Sup-B15 and Molt-4 cells was determined after treatment with DFX and either erastin or ferrostatin-1. (**i**) Western blotting was conducted to assess NRF2, p-NRF2, Ac-NRF2, KEAP1, HO-1, GPX4, and SLC7A11 levels in lysates of Sup-B15 and Molt-4 cells after treatment with DFX and either erastin or ferrostatin-1.

NRF2 participates in a complex regulatory network and plays a pleiotropic role in the regulation of cellular responses, such as apoptosis and autophagy. We assumed that DFX’s effect on leukaemic cell death is also through ferroptosis. Ferroptosis is a programmed cell death related to NRF2 expression and intracellular iron concentration [27]. To examine the involvement of ferroptosis in cell death after DFX treatment, DFX-treated ALL cells were co-introduced with different ferroptosis relevant reagents, such as erastin (ferroptosis inducer) and ferrostatin-1 (ferroptosis inhibitor), to monitor the involvement of ferroptosis in DFX-induced cytotoxicity. The findings revealed adding erastin to DFX-treated Molt-4 and Sup-B15 cells resulted in significantly reduced cell survival and proliferation (Figure 3a,b). Interestingly, a significant elevation of cellular ROS levels (Figure 3c,d) and significantly increased apoptosis and autophagy (Figure 3e–h) were noted compared with those in cells treated with DFX alone. Conversely, the cells co-treated with DFX and ferrostatin-1 had less ROS production and less cell death comparing to those treated with DFX alone. This effect was in proportion to ferrostatin-1 concentration (Figure 3e–h). As predicted, the expression levels of total and active NRF2, as well as its relevant signals [Kelch-like ECH-associated protein 1 (KEAP1), haem oxygenase-1 (HO-1), solute carrier family 7 member 11 (SLC7A11), and GPX4], exhibited significant increases following combined treatment with DFX and erastin; conversely, an observable abolishment was detected in cells co-treated with ferrostatin-1 in both Sup-B15 and Molt-4 cells. These results suggest that ferroptosis was involved in DFX-induced cytotoxicity and cell death, potentially via NRF2-mediated pathways, in ALL cell lines (Figure 3i and Figure S1d).

### 3.4. N-acetylcysteine (NAC) Cannot Reverse the Cytotoxic Effect of DFX on ALL Cells

**Figure 4:**
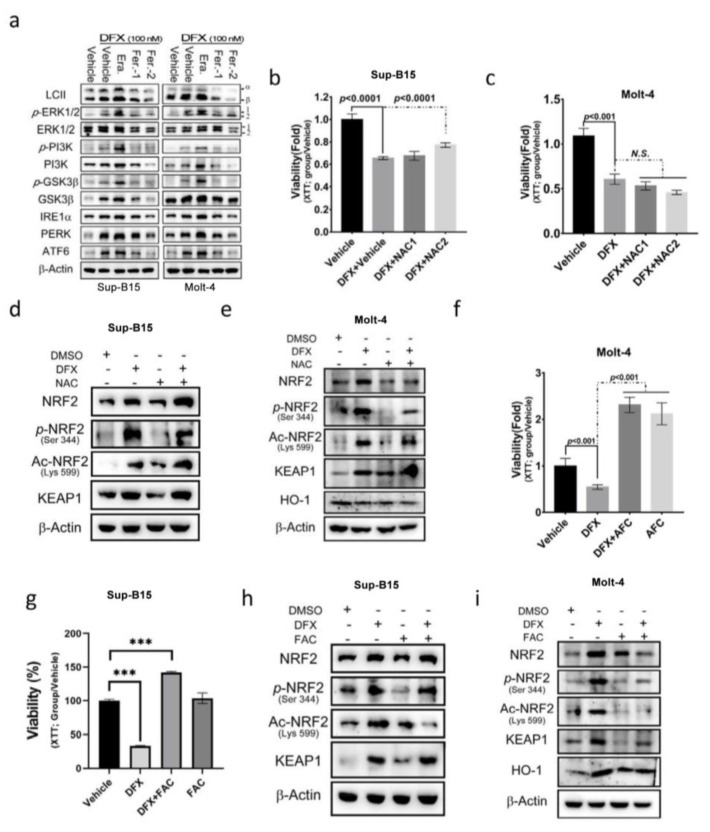
N-acetyl cysteine (NAC) cannot reverse the effect of DFX on ALL cells. (**a**) Sup-B15 and Molt-4 cells were subjected to Western blot analysis to examine the expression of the UPR sensor, and the PI3K, MAPK, and GSK3β signalling pathways following treatment with DFX and either erastin or ferrostatin-1. (**b**,**c**) After 24 h treatment of Sup-B15 and Molt-4 cells with DFX and NAC, cell viability was assessed using an XTT assay. (**d**,**e**) Western blotting was conducted to assess NRF2, p-NRF2, Ac-NRF2, KEAP1, and HO-1 levels in lysates of Sup-B15 cells after DFX and NAC treatment. (**f**,**g**) After 24 h treatment of Sup-B15 and Molt-4 cells with DFX and ferric ammonium citrate (FAC), cell viability was assessed using an XTT assay. ***, *p* < 0.001. (**h**,**i**) Western blotting was conducted to assess NRF2, p-NRF2, Ac-NRF2, KEAP1, and HO-1 levels in lysates of Sup-B15 cells after DFX and FAC treatment.

To gain insight into the signalling mechanisms underlying DFX-induced NRF2 activation, the potential involvement of several key signalling pathways that activate NRF2 activity, including the ERK, PI3K, GSK3, and ER stress pathways, was investigated. The findings revealed that DFX impacts NRF2 activity and cell death by modulating the PI3K, GSK3, and ER stress signalling pathways (Figure 4a and Figure S2a,b). Subsequently, the impact of NAC, an antioxidant, on DFX-treated Sup-B15 and Molt-4 cells was investigated, and cell viability was measured. The results showed that NAC slightly reversed the cytotoxic effect in Sup-B15 cells but not in the Molt-4 cell line (Figure 4b,c). Consistently, NAC treatment marginally decreased phosphorylated NRF2 levels but did not reverse the levels of total and acetylated NRF2 expression in both Sup-B15 and Molt-4 cells (Figure 4d,e and Figure S2c). To investigate the influence of iron concentration on the cytotoxic effect of DFX, excess iron [ferric ammonium citrate (FAC); 20 mM] was introduced to the culture medium, and cell viability was assessed. The results, depicted in Figure 4f–i, demonstrate that leukaemia cells exhibited robust growth under high iron concentrations, and DFX did not exert cytotoxic effects (Figure 4f,g). Moreover, FAC-treated leukaemia cells displayed a significant inhibition of total and active NRF2 levels in Molt-4 cells (Figure 4h and Figure S2d), as well as the inhibition of total and acetylated (Lys599) NRF2 levels in Sup-B15 cells (Figure 4i and Figure S2e). These results highlight the importance of iron overload in cell growth and survival as well as acetylated (Lys599) NRF2 levels in leukaemia cells.

### 3.5. The E1A Binding Protein p300/CREB Binding Protein (p300/CBP) Inhibitor Can Reverse the Effect of DFX on ALL Cells

**Figure 5:**
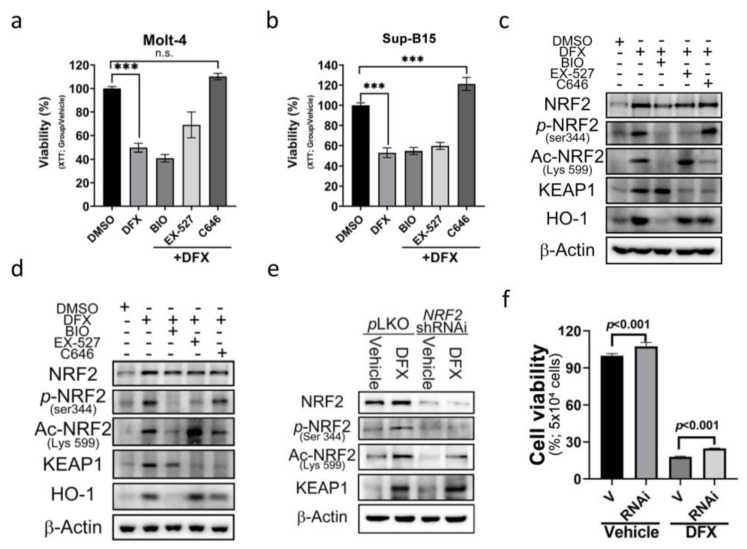
The effect of DFX on ALL cells can be reversed by an E1A binding protein p300/CREB binding protein (p300/CBP) inhibitor. (**a**,**b**) Cell viability was evaluated using an XTT assay after 24 h treatment of Sup-B15 and Molt-4 cells with DFX and different specific chemical inhibitors, including BIO (GSK-3 inhibitor), EX-527 (SIRT1 inhibitor), and C646 (p300/CBP inhibitor). ***, *p* < 0.001. (**c**,**d**) Western blotting was conducted to assess NRF2, p-NRF2, Ac-NRF2, KEAP1, and HO-1 levels in lysates of Sup-B15 and Molt-4 cells after treatment with DFX and different specific chemical inhibitors, including BIO (GSK-3 inhibitor), EX-527 (SIRT1 inhibitor), and C646 (p300/CBP inhibitor). (**e**) Western blotting was conducted to assess NRF2, p-NRF2, Ac-NRF2, and KEAP1 levels in lysates of Sup-B15 cells after treatment with DFX and NRF2 shRNAi. (**f**) Cell viability was evaluated using an XTT assay after 24 h treatment of Sup-B15 cells with DFX and NRF2 shRNAi.

To further dissect the underlying mechanism of acetylated (Lys599) NRF2 in DFX-induced cytotoxicity, three epigenetic regulatory signals were used as potential targets to investigate the role of epigenetics. These regulators included phosphorylase-GSK3, acetyltransferase-p300/CBP, and histone deacetylase-sirtuin 1 (SIRT1). To assess their impact, we utilized specific chemical inhibitors: BIO (GSK-3 Inhibitor), EX-527 (SIRT1 inhibitor), and C646 (p300/CBP inhibitor). The results revealed a significant and remarkable reversal in cell viability upon treatment with C646 (Figure 5a,b). Additionally, a notable downregulation of Ace-Lys599 and KEAP1 was observed upon C646 treatment (Figure 5c,d and Figure S3a); however, no significant changes in the expression levels of total NRF2 were detected. These findings suggest that DFX enhances the activity of p300/CBP acetyltransferase. Through p300/CBP acetyltransferase, Lys599 undergoes acetylation, leading to increased NRF2 activity.

To elucidate the involvement of increased ROS production and NRF2 expression in cell death, short hairpin RNA (shRNA) designed to specifically target and suppress NRF2 expression was employed. NRF2 shRNA was transduced into the Sup-B15 cell line to specifically target and suppress NRF2 expression. The transduction efficacy is depicted in Figure 5e and Figure S3b. Subsequently, cell viability was assessed by adding DFX, and cells transduced with NRF2 shRNA were found to display increased cell viability (Figure 5f). These results suggest that NRF2 indeed plays a role in ALL cell death, and its downregulation contributes to enhanced cell survival in the presence of DFX. 

### 3.6. DFX Treatment Induced Ferroptosis-Mediated Cell Death in the Leukaemia Cells of Patients with ALL

**Figure 6:**
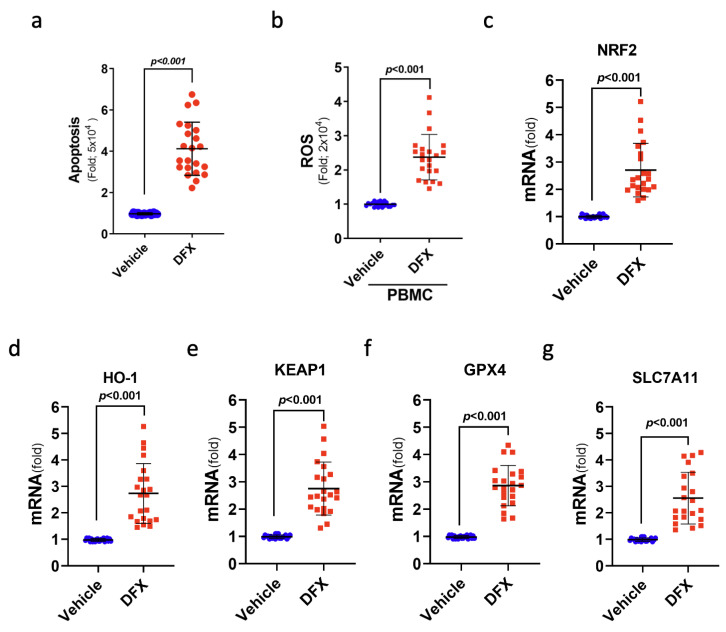
Treatment with DFX induced a type of cell death known as ferroptosis in leukaemia cells from patients with ALL. (**a**) The percentage of apoptotic patients with ALL was determined after DFX treatment. (**b**) Intracellular ROS levels were measured as fold increases in fluorescence relative to the vehicle in ALL peripheral blood mononuclear cells after DFX treatment. (**c**–**g**) The mRNA levels of NRF2, KEAP1, HO-1, GPX4, and SLC7A11 were assessed in ALL patient bone marrow cells (N = 22) after DFX treatment.

To further verify the cytotoxic impact of DFX on leukaemia cells, 22 ALL samples from patients were used to study ferroptosis-related gene expression and cell viability after DFX treatment in a controlled in vitro setting. Notably, DFX treatment yielded substantial increases in cellular death (apoptosis) and intracellular ROS levels in the leukaemia cells of patients with ALL (Figure 6a,b). As anticipated, DFX administration also yielded a significant augmentation in the mRNA levels of genes pertinent to ferroptosis, namely, NRF2, KEAP1, HO-1, GPX4, and SLC7A11, within the leukaemia cells of individuals with ALL (Figure 6c–g). These findings indicate the engagement of ferroptosis-related signals in cases of ALL, highlighting the prospective therapeutic value of anti-ferroptotic interventions in future clinical contexts.

## 4. Discussion

This study provided compelling evidence demonstrating the cytotoxic effect of DFX on leukaemia cells, particularly highlighting its lethal effect on human ALL cells in vitro. Adding DFX to cultured cells, by means of increasement in apoptosis, autophagy, and ferroptosis, led to a reduction in cell viability and proliferation. DFX exerts these cytotoxic effects by augmenting NRF2 activity through the activity of p300/CBP acetyltransferase, in which Lys599 on NRF2 is the acetylating target. Intriguingly, the cytotoxic effect of DFX was abolished when excess iron was added to the culture medium. These findings suggest that DFX therapy holds promise as an effective adjuvant treatment strategy for patients with ALL. Note that while some studies suggest that inhibiting NRF2 can impede tumour growth, our study revealed contrary results in the context of ALL. This discrepancy emphasises the need for further evaluation of the role of NRF2 in ALL treatment. Continued research in this area will provide valuable insights and accelerate the development of novel therapeutic tools for patients with ALL.

Recent studies have revealed that ferroptosis shows promise as a therapeutic strategy for overcoming drug resistance in cancer cells [28]. In addition, ferroptosis can selectively target aggressive cancer stem cells, potentially improving the effectiveness of immunotherapy and overcoming resistance to immunotherapeutic treatments [27,29].

NRF2 participates in a complex regulatory network and performs a pleiotropic regulation role, including ferroptosis. Our study demonstrated that NRF2, especially acetylated NRF2, exerts a cytotoxic effect on leukaemia cells.

The NRF2 signalling pathway is affected by epigenetic alterations that impact its expression, such as DNA methylation and chromatin modifications [21]. For instance, oxidative stress potentially triggers the activity of DNA demethylases, leading to an increase in NRF2 expression and, subsequently, the promotion of chemoresistance. Additionally, the oxidative stress-induced recruitment of O-linked N-acetylglucosamine transferase to the NRF2 promoter mediates the methylation of histone H3 lysine K4, resulting in enhanced NRF2 transcriptional activity [30]. In contrast, methylation of the KEAP1 promoter has been found to reduce KEAP1 levels in lung cancer, a process that is associated with elevated NRF2 expression [22]. NRF2 upregulation in cancer and its role in promoting chemoresistance are influenced by a combination of transcriptional, translational, and epigenetic modifications [31]. However, the specific mechanisms driving these alterations in various cancer types require further investigation.

The p300-CBP coactivator, belonging to the histone acetyltransferase family, is involved in essential biological processes, such as haematopoiesis and embryogenesis [32,33]. Its primary function is to modify lysine residues on histones through acetylation, causing DNA to unwind and facilitating the binding of RNA polymerase II to gene promoter regions. P300-CBP can also acetylate lysine residues on various proteins other than histones [33]. Regarding its interaction with NRF2, the acetylation of lysine residues on NRF2 potentially enhances its binding to the ARE promoter of target genes and regulates gene expression [34,35]. This study focused on the impact of DFX treatment, which led to the increased acetylation of Lys599 on NRF2 [34]. Using inhibitor C646, which targets p300-CBP, acetylation was prevented at this site, and an increase in cell viability was observed [34]. This suggests Lys599 as an NRF2 site potentially acetylated by p300-CBP [34]. Previous studies on mouse NRF2 identified mutations in Lys588 and Lys591 (equivalent to Lys596 and Lys599 in human NRF2) that reduced NRF2-dependent gene expression and abolished the acetylation effect of CBP on NRF2 [34]. Our study’s findings support the applicability of this theory to human cells, although further research is necessary to comprehensively elucidate the specific cellular function and alterations in gene expression resulting from the acetylation of Lys599 on NRF2 [34]. Notably, SIRT1-mediated deacetylation of Lys599 on NRF2 has been observed to lead to the exit of NRF2 from the nucleus and decreased expression of downstream genes, such as NAD(P)H:quinone oxidoreductase 1 and HO-1 [35]. Based on our findings, we propose that the DFX-induced acetylation of Lys599 plays a significant role in inducing cell death in leukaemia cells. Further investigation of the mechanism underlying the DFX-induced activation of p300-CBP is merited.

## 5. Conclusions

In conclusion, DFX demonstrates a potent cytotoxic effect on lymphoblastic leukaemia cells by enhancing NRF2 activity through the acetylation of Lys599 on NRF2 by p300/CBP. However, this effect is attenuated in the presence of high iron concentrations. These findings suggest that DFX is a promising adjuvant therapy for patients with ALL. Administering DFX early in the treatment process rather than after the occurrence of iron overload is crucial. This highlights the importance of timing and DFX incorporation in ALL treatment strategies. Further research is warranted to explore the clinical implications and potential benefits of implementing DFX as an early adjuvant therapy for patients with ALL.

## Data Availability

Data is all contained in the article.

## Supplementary Materials

The following supporting information can be downloaded at: https://www.mdpi.com/article/10.3390/antiox13040424/s1, Figure S1: Results of the quantitative Western blot analysis; Figure S2: Results of the quantitative Western blot analysis; Figure S3: Results of the quantitative Western blot analysis.
