# Supplementary material for: Deferasirox Causes Leukaemia Cell Death through Nrf2-Induced Ferroptosis
Source: Antioxidants (Basel). 2024 Mar 29;13(4):424. doi: 10.3390/antiox13040424 (PMC11047367; doi:10.3390/antiox13040424)
Supplement: Supplementary file 1 [file antioxidants-13-00424-s001.zip › antioxidants-2897251-supplementary.pdf]

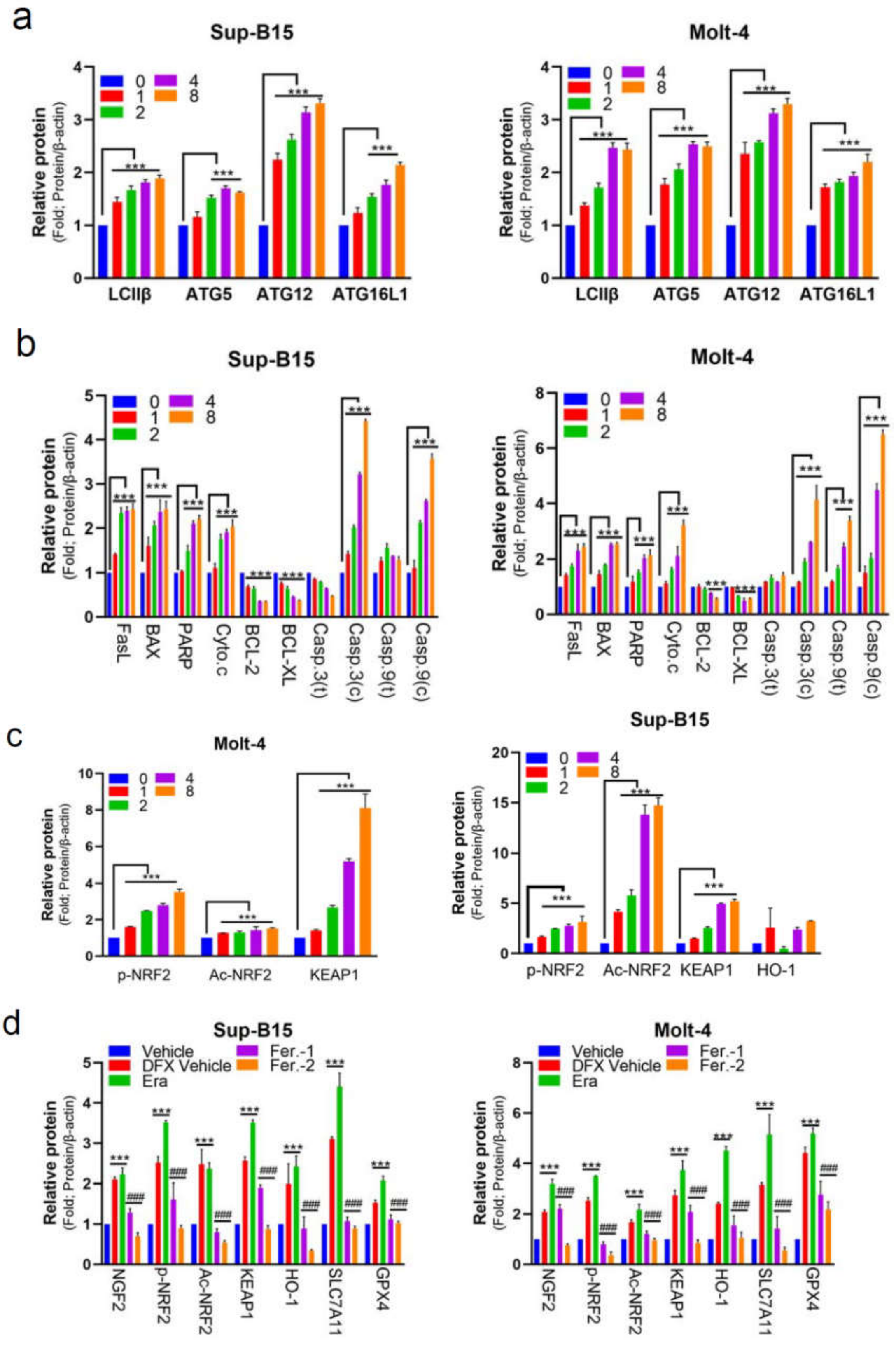

Figure S1. Results of the quantitative Western blot analysis. (a) Western blotting was conducted to assess the levels of autophagic markers (LC3B, ATG5, ATG12, and ATG16L1) in lysates of Sup-B15

and Molt-4 cells after DFX treatment. (b) Western blotting was performed to analyze the levels of apoptotic markers in lysates of Sup-B15 and Molt-4 cells after DFX treatment. (c) Western blotting was conducted to assess the levels of NRF2, p-NRF2, Ac-NRF2, KEAP1, and HO-1 in lysates of Molt-4 cells and Sup-B15 after DFX treatment. (d) Western blotting was conducted to assess NRF2, p-NRF2, Ac-NRF2, KEAP1, HO-1, GPX4, and SLC7A11 levels in anlysates of Sup-B15 and Molt-4 cells after treatment with DFX and either erastin or ferrostatin-1. ***, p<0.001; ###, p<0.001.

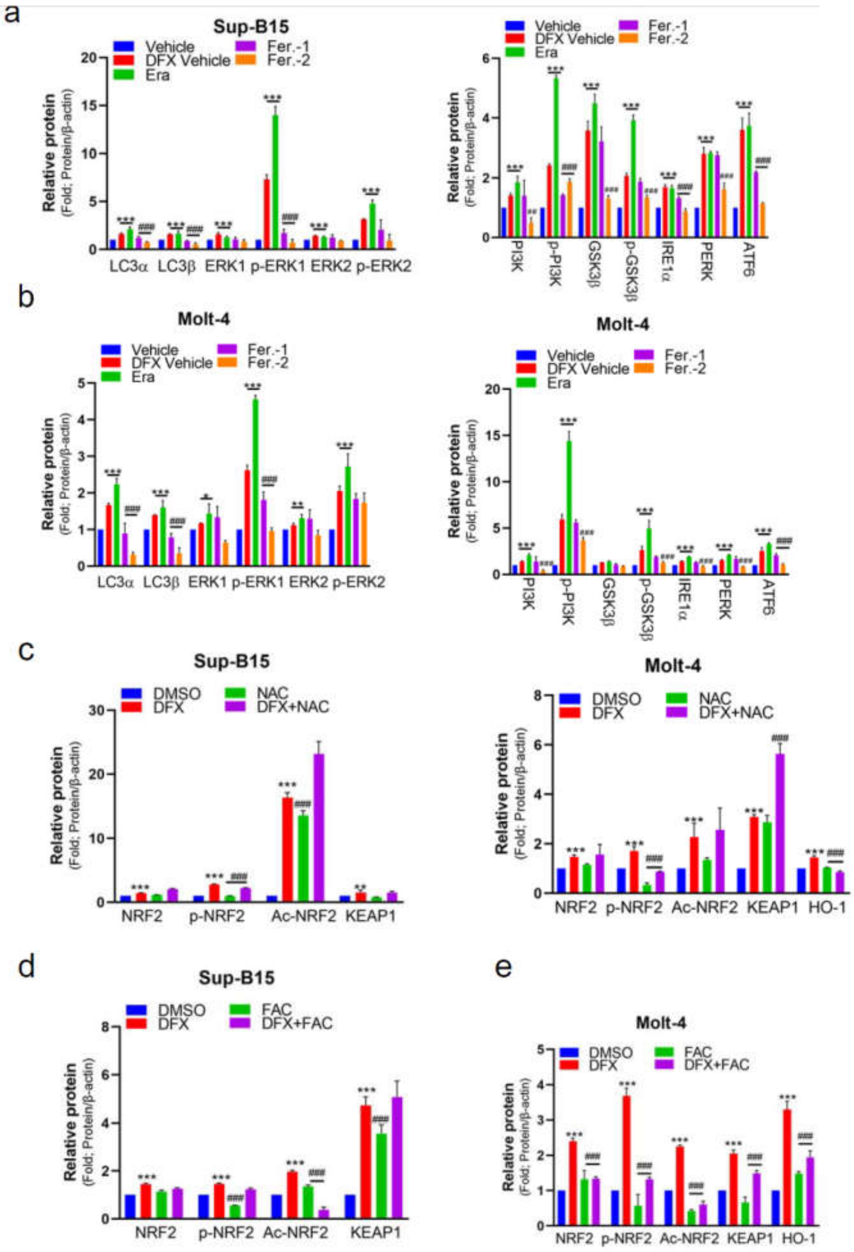

Figure S2. Results of the quantitative Western blot analysis. (a-b) Sup-B15 and Molt-4 cells were subjected to Western blot analysis to examine the expression of the UPR sensor, PI3K, MAPK, and GSK3β signalling pathways following treatment with DFX and either erastin or ferrostatin-1. (c) Western blotting was conducted to assess NRF2, p-NRF2, Ac-NRF2, KEAP1, and HO-1 levels in lysates of Sup-B15 cells after DFX and NAC treatment. (d and e) Western blotting was conducted to assess NRF2, p-NRF2, Ac-NRF2, KEAP1, and HO-1 levels in lysates of Sup-B15 cells after DFX and FAC treatment. *, p<0.05; **, p<0.01; ***, p<0.001, ###, p<0.001

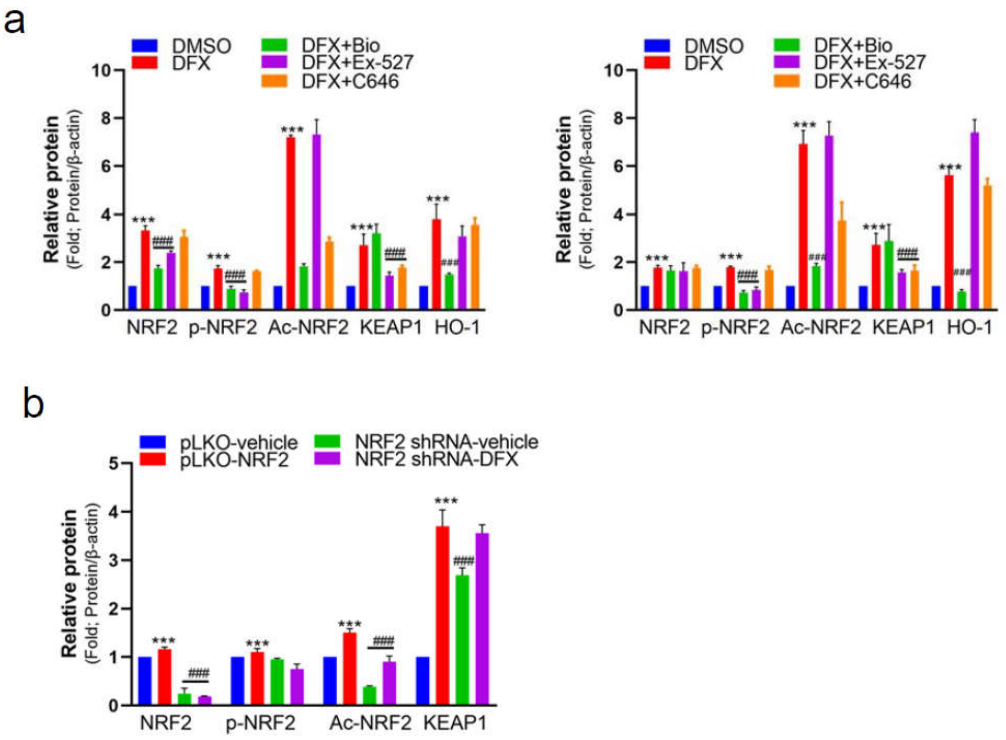

Figure S3. Results of the quantitative Western blot analysis. (a) Western blotting was conducted to assess NRF2, p-NRF2, Ac-NRF2, KEAP1, and HO-1 levels in lysates of Sup-B15 and Molt-4 cells after treatment with DFX and different specific chem-ical inhibitors, including BIO (GSK-3 inhibitor), EX-527 (SIRT1 inhibitor), and C646 (p300/CBP inhibitor). (b) Western blotting was conducted to assess NRF2, p-NRF2, Ac-NRF2, and KEAP1 levels in lysates of Sup-B15 cells after treatment with DFX and NRF2 shRNAi. ***, p<0.001; ###, p<0.001.
